# Boundary conditions for free A-DNA in solution and the relation of local to global DNA structures at reduced water activity

**DOI:** 10.1007/s00249-015-1110-1

**Published:** 2016-02-13

**Authors:** Dietmar Porschke

**Affiliations:** Max Planck Institut für biophysikalische Chemie, 37077 Göttingen, Germany

**Keywords:** Electric dichroism, Rotational diffusion, Worm-like chain, Rise per base pair, DNA state diagram, Condensation

## Abstract

Because of repeated claims that A-DNA cannot exist without aggregation or condensation, the state of DNA restriction fragments with 84–859 bp has been analyzed in aqueous solutions upon reduction of the water activity. Rotational diffusion times *τ*^d^ measured by electric dichroism at different water activities with a wide variation of viscosities are normalized to values *τ*^c^ at the viscosity of water, which indicate DNA structures at a high sensitivity. For short helices (chain lengths ${\ell}$ ≤ persistence length *p*), cooperative formation of A-DNA is reflected by the expected reduction of the hydrodynamic length; the transition to the A-form is without aggregation or condensation upon addition of ethanol at monovalent salt ≤1 mM. The aggregation boundary, indicated by a strong increase of *τ*^c^, is shifted to higher monovalent salt (≥4 mM) when ethanol is replaced by trifluoroethanol. The BA transition is not indicated anymore by a cooperative change of *τ*^c^ for ${\ell}$ » *p*; *τ*^c^ values for these long chains decrease upon reduction of the water activity continuously over the full range, including the BA transition interval. This suggests a non-cooperative BC transition, which induces DNA curvature. The resulting wide distribution of global structures hides changes of local length during the BA transition. Free A-DNA without aggregation/condensation is found at low-salt concentrations where aggregation is inhibited and/or very slow. In an intermediate range of solvent conditions, where the A-form starts to aggregate, a time window remains that can be used for analysis of free A-DNA in a quasi-equilibrium state.

## Introduction

The transition of B- to A-DNA is observed in vitro under the special conditions of strongly reduced water activity (Franklin and Gosling 1953). Partly because of these unusual conditions, A-DNA is not always accepted as an entirely natural form of DNA, although the A-form of DNA has been demonstrated to exist in the natural environment of protein–DNA complexes (Cheetham and Steitz 1999; Jacobo-Molina et al. 1993; Jones et al. 1999; Kiefer et al. 1998; Lu et al. 2000). A special role for A-DNA has been indicated recently by its detection in an extremophile (DiMaio et al. 2015), living under extreme conditions of high temperature and low pH. In vitro, the water activity is commonly reduced for induction of A-DNA by addition of alcohol (Brahms and Mommaerts 1964). It is well known that addition of alcohol induces precipitation of DNA (Girod et al. 1973; Shapiro 1981). In fact, formation of A-DNA has often been observed together with aggregation. Thus, the conclusion appears to be obvious and has been presented by many different authors that generation of A-DNA is always coupled with aggregation or condensation (Herbeck et al. 1976; Nishimura et al. 1985; Zimmerman and Pheiffer 1979).

This view was recently presented again by Hormeno et al. (2011), based on single-molecule experiments using force-extension with optical tweezers. Hormeno et al. claimed that “condensation prevails over B-A transition in the structure of DNA at low humidity” and that “no contour length change compatible with a cooperative transition between the A and B forms” was found. The question of an inherent coupling between the BA transition and aggregation or condensation is not only of academic but also of practical interest. Such coupling is essential for the interpretation of both equilibrium and kinetic data.

Most of the available data on the BA transition have been collected by Ivanov et al. (e.g. Ivanov and Krylov 1992; Ivanov et al. 1974) at low-salt concentrations based on experimental evidence that aggregation is avoided at low-salt conditions. It is remarkable that the authors claiming a crucial role of aggregation for the BA transition usually do not consider the arguments of the authors supporting the existence of free A-DNA. Are these arguments not convincing enough? Basic physical chemistry tells that aggregation of highly charged objects is suppressed at low-salt conditions, but experimental details should be examined carefully. Aggregation is dependent on many different parameters and experimental techniques for unequivocal exclusion and/or quantification of aggregation are usually elaborate and require experience. This seems be the reason why these techniques have been applied only to a limited extent.

Analytical ultracentrifugation was used by Herbeck et al. (1976) for calf thymus DNA at 0.2 mM salt: at 70 % EtOH, the sedimentation coefficient was identical to that in water, whereas at 80 % EtOH a strong increase indicated aggregation. The authors suggest that “aggregation stabilizes the A form through lateral interactions between the helices.” In addition, they comment that “it seems to be apparent that DNA cannot even exist in the A-form as a single isolated chain in solution”. Potaman et al. (1980) compared in a subsequent analysis of calf thymus and T7 phage DNA at 0.5 mM NaCl the changes of the sedimentation coefficients and the CD spectra upon addition of EtOH in the BA transition range. They found aggregation of DNA at high EtOH percentage. However, the change of sedimentation coefficients was still small at a degree of ~66 % transition to the A-form assigned by their CD data. This is the basis for their conclusion that “aggregation is not a necessary condition for the B → A transition.”

Ivanov’s group (1978) determined the degree of DNA orientation in a flow within the interval of the BA transition and found nearly identical orientation below and above the transition; they conclude “that there is no aggregation in the course of formation of the A form.” However, some change of the flow-induced orientation is expected to result from the length reduction during the BA transition. Thus, the question is raised again: are the arguments in favor of A-DNA without aggregation at low salt convincing enough? The molecular interactions contributing to the BA transition are of sufficiently general interest for an independent careful analysis.

Ivanov et al. claimed repeatedly that aggregation can be suppressed by using trifluoroethanol instead of ethanol for reduction of the water activity. However, direct experimental data in support of this statement apparently have not been published. Because of this gap in the literature, the present investigation has been extended to the case of trifluoroethanol.

Structures in solution and potential aggregation may be analyzed at a particularly high sensitivity by measurements of rotational diffusion. Electro-optical techniques are most attractive for the analysis of DNA because the high optical anisotropy of DNA together with the high electrical anisotropy at low-salt concentrations used for A-DNA provides an optimal basis for accurate results (Fredericq and Houssier 1973; Porschke and Antosiewicz 2007). Some reports on electro-optical measurements of A-DNA have been published (Charney and Chen 1987; Wu et al. 1981), partly with contradictory results. In the present investigation, the BA transition is analyzed in more detail over a range of different chain lengths and with special emphasis on the aggregation/condensation controversy. The experimental data demonstrate the state of DNA in solution and the boundary conditions for free A-DNA at a high accuracy and reliability. Moreover, the results provide new information about the transition from B- via C- to A-DNA and explain the nature of the problem with detection of length changes during the BA transition described by Hormeno et al. (2011).

## Materials and methods

DNA fragments were prepared as described previously (Porschke 1991b). All DNA fragments were blunt ended. Absolute ethanol for analysis was obtained from Merck (Darmstadt, Germany). 2,2,2-Trifluoroethanol ReagentPlus ≥ 99 % was from Sigma; lot # MKBQ9888V was used exclusively because this lot had a sufficiently low UV absorbance. The standard buffer B contained 1 mM NaCl, 1 mM Na-cacodylate pH 7.0 and 0.2 mM EDTA. A dilution of buffer B by a factor of 10 is denoted buffer A. Addition of 2 and 10 mM NaCl to buffer B resulted in buffers C and D, respectively. Ethanol or trifluoroethanol was always added as the last component, except for small final additions of water to arrive at a well-defined total volume in small volumetric flasks. The alcohol content at the stage of mixing was controlled by weighing. The solutions had to be degassed before exposure to electric field pulses under vacuum, which induces some reduction of the alcohol content. For this reason, the exact alcohol content during the measurements was determined after completion of the field jump experiments by evaluation of the density using a DMA60/602 (Anton Paar, Graz, Austria). UV spectra of samples were measured before and after exposure to electric field pulses directly in the field jump cell in a PerkinElmer Lambda 17 spectrophotometer. Conductivities were controlled routinely.

The absorbance of the DNA samples at 260 nm and 1 cm path length was in the range of 0.05–0.2, corresponding to helix concentrations in the range of 5–150 nM, depending on the chain length. The measurements started usually ~30 min after mixing. At high values of the alcohol content (given in volume% ≡ ‘Vol%EtOH’ or ‘Vol%TFE’), a dependence of the dichroism curves on the time after mixing was observed. This effect appeared in the range, where DNA association was indicated by other criteria like turbidity as well.

The electric dichroism was measured as described previously (Grünhagen 1974; Porschke 1980). A novel construction of optical windows was used in the cells for these measurements (publication in preparation). Dichroism transients were routinely recorded at 248 nm for parallel orientation of the polarized light with respect to the field vector. Control experiments at the magic angle at maximal electric field strengths demonstrated the absence of field-induced denaturation. The reduced linear dichroism is given as $\xi = 1.5*\Delta E_{||} /\bar{E}$, where $\Delta E_{||}$ is the stationary change of the absorbance at parallel orientation of polarized light with respect to the field vector and $\bar{E}$ is the isotropic absorbance. The temperature used for the measurements was 20 ± 0.1 °C. Time constants were fitted to dichroism decay curves by standard programs (Diekmann et al. 1982; Porschke and Jung 1985) developed previously. The time constants were always measured over a range of different electric field strengths. As described previously (Porschke 1986), there is usually an increase of these time constants with increasing field strength. Linear regression of these data provided time constants at zero electric field strength.

Because the viscosity of water alcohol mixtures shows a strong dependence on the alcohol content, the time constants had to be converted to a standard viscosity for comparison. Thus, all time constants given in figures or elsewhere are converted to the viscosity of water at 20 °C by the standard viscosity conversion factor. This correction is indicated by the superscript “c”, e.g., *τ*^c^. Densities and viscosities for water–ethanol mixtures were taken from the CRC Handbook of Chemistry and Physics. Densities and viscosities for water–trifluoroethanol (TFE) mixtures were obtained from Esteve et al. (1995) and Olive et al. (1996). Polynomial fitting and interpolation was used for evaluation of vol%EtOH or vol%TFE from measured densities and for determination of viscosity values at given vol% EtOH or vol%TFE.

## Results

### Rotational diffusion time constants

**Fig. 1:**
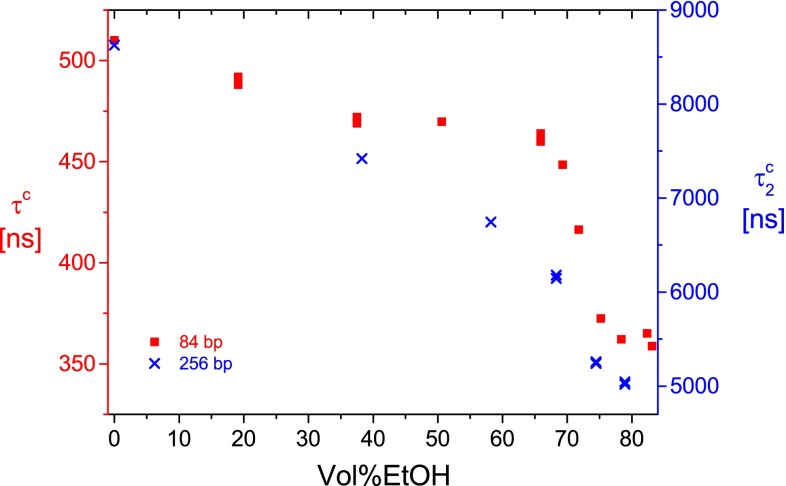
Dichroism decay time constants of 84- and 256-bp DNA fragments at 20.0 °C in buffer A as a function of the ethanol content in vol%. The scale on the *left side* represents the single exponential time constants *τ* ^c^ for the 84-bp fragment; the scale on the* right side* represents the slow component $\tau_{2}^{\text{c}}$ of double exponential fits for the 256-bp fragment

DNA fragments with chain lengths in the range up to ~100 bp are below the persistence length and thus can be considered as rigid rods at a reasonable approximation. In this limit, the dichroism decay can be fitted by single time constants. Measurements for a fragment with 84 bp in buffer A as a function of the ethanol content (Fig. 1) demonstrate a strong decrease of the time constants normalized to the viscosity of water *τ*^c^ in a narrow range of the ethanol content, indicating the BA transition. Some decrease of the time constants is observed already in the range of vol%EtOH below the BA transition, but this effect remains relatively small. For comparison, data obtained for a fragment with 256 base pairs are shown, scaled upon the data for the shorter fragment. Fitting of the dichroism decay of the longer fragment requires two time constants. The second time constant $\tau_{2}^{\text{c}}$ reflects the overall hydrodynamic dimensions and is shown in Fig. 1. For the 256 bp fragment, there is again a cooperative decrease in the range of the BA transition, but the amplitude of this decrease is small compared to the relatively large decrease in the preceding vol%EtOH range.

**Fig. 2:**
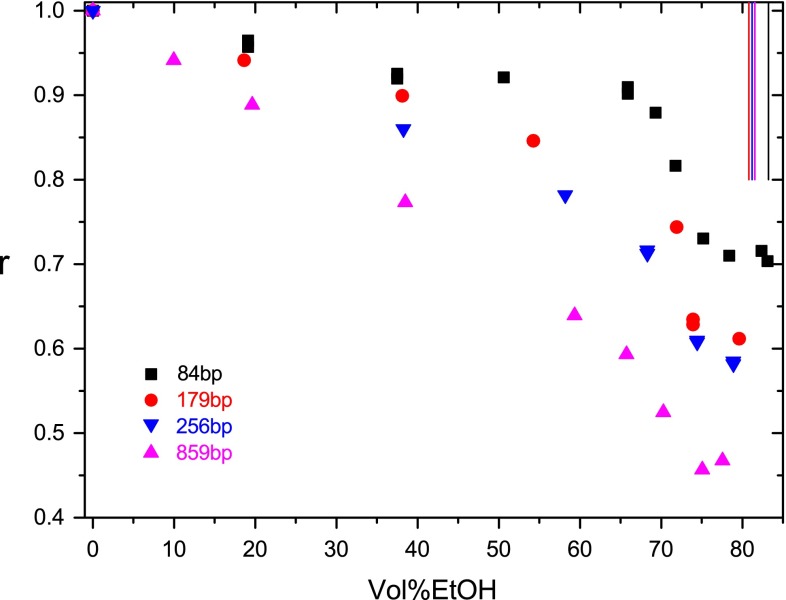
Ratio *r* of the dichroism decay time constants measured at a given ethanol content to that measured without ethanol addition at 20 °C in buffer A as a function of the ethanol content in vol% for different DNA fragments. The *vertical lines* (*color code* as in the *symbols*) represent approximate values for the limit ethanol content, where aggregation starts to prevail

**Fig. 3:**
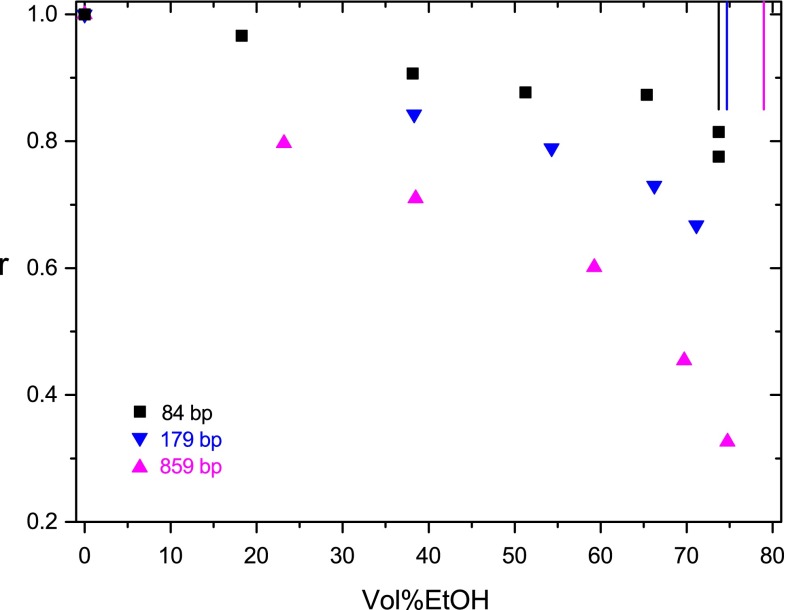
Ratio *r* of the dichroism decay time constants measured at a given ethanol content to that measured without ethanol addition at 20 °C in buffer B as a function of the ethanol content in vol%. The *vertical lines* (*color code* as in the *symbols*) represent approximate values for the limit ethanol content, where aggregation starts to prevail

A further shift in the dependence of the time constant on the water activity is observed for the fragment with 859 bp. In this case, the BA transition is not reflected anymore by a clear cooperative decrease of the time constant in the expected narrow range of the water activity. Instead, there is an overall continuous decrease of the time constant extending from 0 to 80 vol%EtOH. This is shown in Fig. 2 in terms of ratios of time constants measured in buffer A at given vol%EtOH relative to the respective time constants found in the absence of ethanol. The dependence of the ratio *r* on the ethanol content for different DNA fragments shows a continuous decrease of the change Δ*r*_A_ in the BA transition range with increasing chain length, whereas the change Δ*r*_C_ in the range below the BA transition increases. Corresponding data at a higher salt concentration (buffer B) are shown in Fig. 3. The dependence is similar to that found in buffer A, but the limit of aggregation is shifted to lower vol%EtOH at the higher salt concentration. Thus, the main part of the BA transition is hidden in buffer B even for the 84-bp fragment by aggregation effects.

### Aggregation

**Fig. 4:**
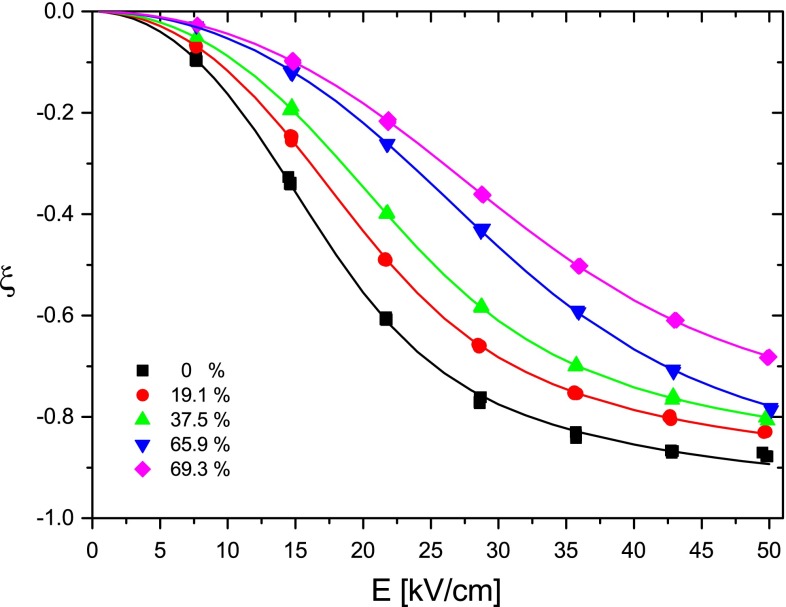
Reduced linear dichroism ξ for the 84-bp fragment in buffer A at 20 °C as a function of the electric field strength E at different ethanol contents in vol%. The *lines* show least squares fits by the saturating induced dipole model; parameters are given in Fig. 5

It is well known that DNA can be condensed, aggregated, or precipitated by addition of ethanol (Bloomfield 1996; Girod et al. 1973; Lang 1969; Shapiro 1981). Precipitation is the final stage of a process going in different reaction steps, which are affected by various parameters, including the ionic conditions, the DNA concentration, and chain length. Because the main goal in the present context is avoidance of these “perturbations”, observations on aggregation effects are presented here within these limits and without a systematic investigation. Electro-optical time constants are very sensitive indicators of aggregation, particularly in the range of chain lengths ≤ persistence length. In the case of the 84-bp fragment in buffer A, the first indication for aggregation appeared at 75.17 vol%EtOH in the form of a slow component with $\tau_{2}^{\text{c}} \ge 20\,\upmu{\text{s}}$ in the dichroism decay. The relative amplitude of this component was 1.7 % in the range of 50 kV/cm, where the overall degree of orientation approaches a high level (cf. Fig. 4) and thus the amplitude percentage reflects the population percentage at a sufficiently high accuracy. At lower field strengths, the slow relative amplitude is higher due to a relatively high polarizability of the aggregates. At this stage, the decay time constants $\tau_{1}^{\text{c}}$ of the DNA monomers can still be measured at a high accuracy because of their large relative amplitude (>90 % at ~50 kV/cm). When the EtOH content is increased, the relative amplitude of the slow component increases. A test experiment revealed that the slow amplitude observed in the range of vol%EtOH > 75 increases slowly with time, indicating a slow association reaction [cf. (Hillen and Wells 1980)].

These observations demonstrate that there is a transition range, where part of the DNA starts to be aggregated, but the free form of A-DNA can still be characterized at a high accuracy. When the EtOH content is increased further under the conditions of the present experiments, limit values of vol%EtOH appear, beyond which aggregation is dominant and the characterization of free A-DNA requires a higher accuracy and more efforts. These limit values are indicated by vertical lines in Figs. 2 and 3.

In the vol%EtOH-range with initial aggregation, measurements of UV spectra as a function of time after EtOH addition show the existence of an induction period with relatively small and slow changes of the absorbance followed by a period with relatively fast and large absorbance changes. The existence of such induction periods may be used for the characterization of A-DNA in a transient state under limit conditions.

Separate amplitudes with time constants larger by factors of ~100 than those of the “free” state are also observed at vol%EtOH ≥ 80 for the fragments with 179 and 256 bp in buffer A. The analysis is more difficult for still longer fragments, because the broad range of wormlike chain configurations of long chains leads to a broad range of time constants. The time constants observed for the fragment with 859 bp in buffer A are larger in the range vol%EtOH ≥ 80 than those found at lower ethanol contents, but the increase is not nearly as much as observed for the shorter fragments. It is conceivable that there is intramolecular condensation of long fragments, at least partly, in contrast to the intermolecular aggregation indicated by the present results for relatively short fragments. These phenomena should be analyzed in more detail, as intra-molecular condensation effects may be distinguished from inter-molecular aggregation on the basis of their kinetics (Porschke 1984, 1991a).

When the salt concentration is increased from 0.24 mM (buffer A) to 2.4 mM (buffer B), aggregation effects appear at lower ethanol contents (cf. Fig. 3). Aggregation effects and the BA transition are observed in about the same range of ethanol content in buffer B for the restriction fragments used in the present investigation. Experiments on the 84-bp fragment in buffer D demonstrate a further decrease of the aggregation limit to ~66 vol%EtOH at the ionic strength of 12.4 mM.

### Evaluation of the rise per base pair

The fragment with 84 bp in buffer A shows a cooperative reduction of the hydrodynamic length upon the transition to the A-form. Because the 84-bp fragment is rather close to the limit of a rigid rod, the experimental time constants may be used for an evaluation of the rise per base pair. As demonstrated by comparison of values for different limit models (see below), DNA bending effects should be considered for an increased accuracy. The length dependence of the *τ*^d^ values for blunt-ended restriction fragments in buffer A without ethanol indicates a persistence length of 2000 Å and a hydrodynamic helix radius of 12 Å.

The dichroism decay time constant for the 84-bp fragment found in the absence of ethanol $\tau^{\text{d}} = 510 \;{\text{ns}}$ decreases to *τ*^c^ = 362 ns upon formation of A-DNA (at vol%EtOH > 78; cf. Fig. 1). The model for rigid rods by Tirado and Garcia de la Torre (1980) predicts a time constant of 532 ns for a rise per base pair of 3.4 Å and a radius of 12 Å. Application of the wormlike chain correction by Hagerman and Zimm (1981) provides a value of 515 ns for a persistence length of 2000 Å. This comparison shows that the 84-bp fragment is rather close to a rigid rod, but the time constants are already affected by the flexibility/curvature to some degree. A corresponding calculation assuming the same helix radius and persistence length with a rise per base pair of 2.94 Å provides the values 370 and 361 ns for the rigid rod and the flexible rod, respectively. Thus, the experimental *τ*^c^ = 362 ns for the 84-bp fragment at vol%EtOH > 78 indicates an average rise per base pair of 2.94 Å for A-DNA in buffer A (estimated accuracy ±0.05 Å).

The experimental time constant for the 84-bp fragment in buffer A at 65.9 Vol %EtOH, before the onset of the BA transition, is 462 ns. Using again a helix radius of 12 Å and a persistence length of 2000 Å, this time constant corresponds to a rise per base pair of 3.25 Å—an approximate experimental value for C-DNA under the given conditions.

### Linear dichroism and its limit value

The field-induced orientation of DNA double helices is reflected by the reduced linear dichroism ξ. The magnitude of ξ shows the degree of DNA alignment, which increases with the electric field strength E. The dependence of ξ on E is described by an orientation function (Fredericq and Houssier 1973), corresponding to a Boltzmann function, which can be used to evaluate the limiting linear dichroism ξ_∞_ at infinitely high electric field strength and the electrical parameters of the DNA. ξ_∞_ is a measure of the base pair orientation with respect to the helix axis and can be used to derive the average angle of the transition dipoles of the bases with respect to the helix axis.

**Fig. 5:**
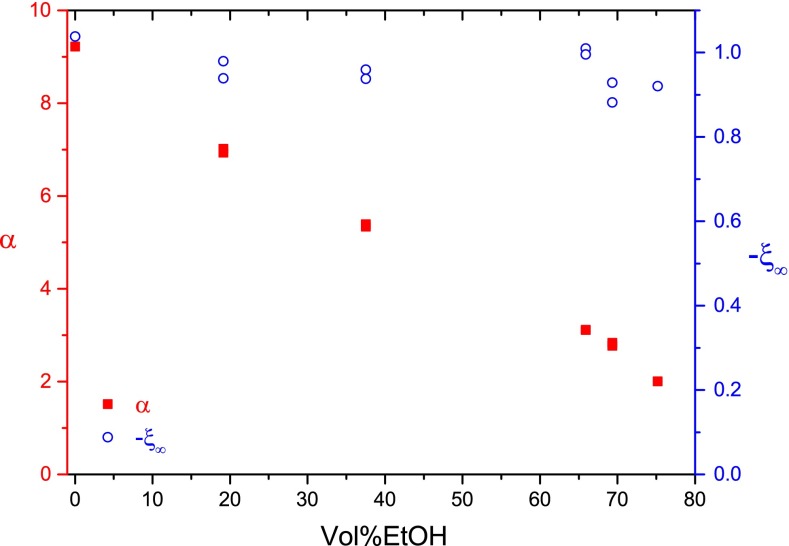
Polarizability α (10^−33^ Cm^2^ V^−1^) and limiting value of the reduced linear dichroism ξ_∞_ for the 84 bp fragment in buffer A at 20 °C as function of the ethanol content in vol%

The reduced linear dichroism ξ as a function of the field strength E for the 84-bp fragment in buffer A is given in Fig. 4 at different vol%EtOH. Fitting of these data by the orientation function for a saturating induced dipole (Diekmann et al. 1984) provides the following results (cf. Fig. 5): (1) the increase of the ethanol content induces a strong decrease of the polarizability; (2) the limit reduced dichroism ξ_∞_ remains constant within experimental accuracy. It is remarkable that the transition from the B- to the A-form is not associated with a clear change of the limit dichroism. The dichroism parameters for the other DNA fragments are similar. The present results on the limit linear dichroism of A-DNA are consistent with those of Charney and Chen (1987), but are different from those reported by Wu et al. (1981). The ξ_∞_ values indicate that the bases are inclined at an average angle of ~70° with respect to the helix axis both for the A- and the B-form under the given experimental conditions.

### Reduction of water activity by addition of trifluoroethanol

**Fig. 6:**
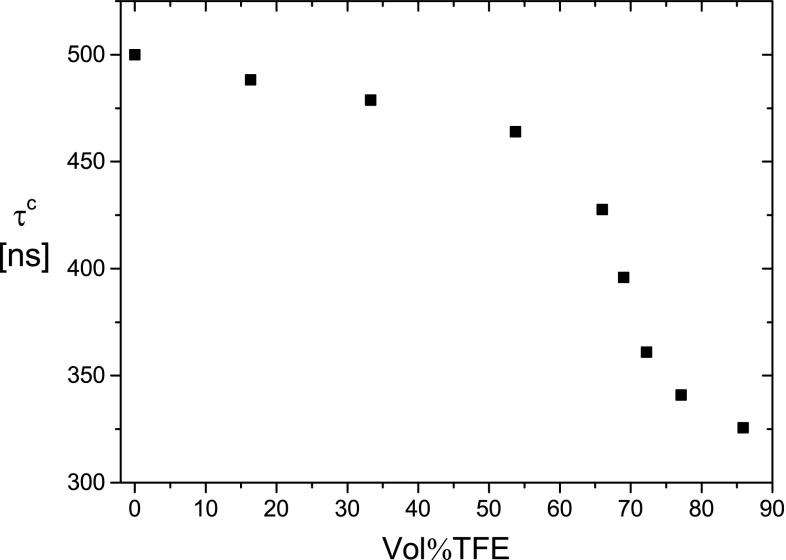
Dichroism decay time constant *τ* ^c^ of the 84-bp DNA fragment at 20.0 °C in buffer C (ionic strength 4.4 mM) as a function of the trifluoroethanol content in vol%

The experimental data for DNA in water–trifluoroethanol mixtures were measured and analyzed as described for the case of ethanol. The results are very similar, but the range of experimental conditions without aggregation and precipitation is extended. Thus, free A-DNA can be observed up to higher salt concentrations. This is demonstrated for the case of the DNA fragment with 84 bp at the Na^+^-concentration of 4.4 mM in Fig. 6.

A more extensive characterization of DNA samples in various water–trifluoroethanol mixtures revealed special effects, which are not directly related to the main subject of the present investigation. These effects will be described elsewhere.

## Discussion

The data obtained by electro-optical measurements provide information mainly on the global structure of DNA restriction fragments in solution. It is well known that this global structure is determined both by the flexibility of the double helix and inherent long-range structures imposed by the sequence. Separation of these effects, for example in terms of a dynamic and a static persistence length (Trifonov et al. 1987), is not simple. A very sensitive measure of the global structure is the rotational time constant, which has been determined in the present investigation from the decay of the field induced linear dichroism. A potential perturbation of the experimental data resulting from field-induced stretching of the global structure by the applied electric field has been avoided by extrapolation of the data to zero electric field strength.

### B → C → A transitions

The time constants observed for the shortest fragment with 84 bp are particularly useful for demonstration of structure changes upon reduction of the water activity. Because the length of this fragment is below the persistence length, the interpretation of the data is relatively simple. A very clear effect is the cooperative reduction of the time constant in the range around 73 vol%EtOH, indicating the BA transition. In addition, there are two other effects that can be identified: (1) a decrease of the time constant in the vol%EtOH range below the BA transition and (2) an increase appearing at higher vol%EtOH than the BA transition. The latter one is expected in this regime of high vol%EtOH and represents association of DNA strands, which finally leads to precipitation. The decrease below the BA transition has not been described yet. A comparison with observations presented in the literature indicates that it represents a BC transition. C-DNA was identified during early studies of DNA structure by fiber diffraction (Arnott and Selsing 1975; Saenger 1984; Zimmerman and Pheiffer 1980). Various experimental data show that the BC transition is non-cooperative (Brahms et al. 1973), which is consistent with the present observations. C-DNA is reported to have an axial rise per residue of 3.31 Å with base pairs slightly tilted by 8° and to repeat itself exactly after three turns with 28 bp. The rise per base pair obtained in the present investigation at 65.9 vol%EtOH of 3.25 Å is in close agreement with the literature value for C-DNA and is clearly different from the value of 3.4 Å established for B-DNA (Bloomfield et al. 2000; Saenger 1984). Thus, reported properties of C-DNA including the rise per base pair and non-cooperative formation at reduced water activity agree with the present observations and the assignment seems to be sufficiently clear.

Van Dam and Levitt (2000) concluded from a combination of NMR, X-ray diffraction, and model building that B and C forms of DNA have two distinct nucleotide conformations, corresponding to the BI and BII conformations known from oligonucleotide crystals. The proportion of the BII conformation is higher in the C form than in the B form and the BC transition corresponds to BI-BII conformational changes of nucleotides. Tisne et al. (1998) observed helix curvature associated with BII conformations by solution NMR in a DNA hexamer. Thus, the decrease of the rotational time constants upon reduction of the water activity in a broad range of vol%EtOH is consistent with DNA curvature associated with a non-cooperative BC transition.

Because of the close similarity of B- and C-DNA, the question has been raised whether definition of a separate helix form is justified. Such doubts may continue to exist as long as a high-resolution crystal structure of C-DNA has not been found yet. Probably, C-DNA crystals have not been found because of sub-optimal crystal packing effects, e.g., resulting from curvature. Usually the assignment of structures in solution is supported by CD spectroscopy. Differences in CD spectra between B- and C-DNA have been reported. A “schematized” CD spectrum for C-DNA was presented by Ivanov et al. (1973), for example, but a different assignment was given by Bokma et al. (1987). Apparently an unequivocal deduction of a reference CD spectrum for C-DNA is difficult (Bokma et al. 1987). Under these special conditions, rotational diffusion time constants obtained from electro-optical measurements are very useful for the characterization of DNA structures *in solution* because of their high sensitivity. Changes of structure can be identified at a high accuracy, also those induced in solvent systems other than simple aqueous environments. It may be noted here that the solvent conditions explored in the present investigation cover a range which may be closer to that in living cells than a simple aqueous environment.

### Hiding of BA transition

A special phenomenon in the set of hydrodynamic data obtained in the present investigation is the apparent disappearance of the BA transition when the chain length is increased. The hydrodynamic data demonstrate a clear decrease of the cooperative amplitude associated with the BA transition with increasing DNA chain length, whereas the amplitude appearing in the range of vol%EtOH below the BA transition increases. The change of the time constant in the BA transition range is quite large for the fragment with 84 bp, but is hardly visible anymore at 859 bp. These data may induce the suspicion that the B–A transition is suppressed at increased chain lengths, but other experimental approaches like CD titrations (Hillen and Wells 1980), indicating the local structure, never provided any evidence for such an unusual effect. Thus, the BA transition does not disappear at high chain lengths, but is hidden in the hydrodynamic data by the BC transition and the wide variation of the DNA configurations at high chain lengths. This does not reduce the value of the rotational time constants, which are particularly sensitive, but illustrates the difficulty to separate different effects in the hydrodynamics of long chains. It should be emphasized that the effects described here, appearing below the aggregation limit, cannot be attributed to condensation because a total stepwise collapse is characteristic of condensation (Bloomfield 1996).

Knee et al. (2008) reported evidence for a “sequential mechanism for the A-to-B transition in DNA”. They used ultraviolet resonance Raman spectroscopy, suggesting that the “transition involves a series of intermediate forms between A and B. Cooperative and distinct transitions were observed for the bases and the sugars.” The present hydrodynamic data do not show evidence for such “cooperative distinct transitions” in the intermediate range of the water activity; cooperativity is found only in the standard range of the BA transition. The preceding change assigned to a BC transition is extended over a broad range of water activity and is without indication for cooperativity, in agreement with early observations (Brahms et al. 1973).

### Condensation and aggregation

Both the BA and BC transition induced by addition of ethanol can be studied in solution only at low-salt concentrations because an increase of the salt induces condensation and aggregation. Condensation and aggregation are associated with particularly large changes of electro-optical effects and, thus, can be easily distinguished from other phenomena. The data obtained in the present investigation demonstrate the range of conditions, where the BA transition can be characterized without perturbations by condensation or aggregation. The claim (Hormeno et al. 2011) that “condensation prevails over B-A transition in the structure of DNA at low humidity” is correct for the conditions of their experiments but is not correct in general. The experimental data supporting this claim were obtained at a too high salt concentration. It is well known that the tendency for condensation and aggregation is reduced at low-salt concentrations. Furthermore, the absence of a “contour length change compatible with a cooperative transition between the A and B form” is characterized here by measurements of the chain length dependence as a special effect in the range of high chain lengths used by Hormeno et al.: changes of the contour length are hidden in the broad distribution of wormlike chain configurations. Previously published experiments on a 70 bp poly[d(AT)] sample (Jose and Porschke 2004) demonstrated already the expected length reduction upon the BA transition without perturbation by condensation or aggregation. However, a detailed analysis of the various factors over a broad range of different chain lengths had not been presented yet. As described independently in a recent publication (Vafabakhsh and Ha 2012), the single-molecule approach by force extension experiments can be applied only in a limited range. Thus, electro-optical techniques continue to be very useful for the analysis of DNA structures in solution. Electro-optics is particularly powerful in the range of low-salt concentrations where processes like the BA transition of DNA can be analyzed without perturbations.

The results of the present investigation were obtained over a range of different salt concentrations, but the analysis was restricted to buffers with Na^+^ cations. Some results reported in the literature demonstrate that the nature of the cation can have a significant effect (Vorlickova et al. 1982). A clear influence of a detail in the solvent composition is demonstrated here by the comparison of ethanol with trifluoroethanol for reduction of the water activity. Thus, details of the solvent composition should be considered in more detail.

For future investigations, it may be of interest that intermolecular aggregation and precipitation are relatively slow processes at the low-DNA concentrations, which are sufficient for various spectroscopic types of analysis. Thus, there is a time window available for studies of A-DNA even in the critical range of conditions, where aggregation and precipitation cannot be avoided at long times.
